# A Case Report of Acute Acalculous Cholecystitis and Acute Hemorrhagic Cystitis due to Salmonella Typhi

**DOI:** 10.1155/2014/758583

**Published:** 2014-08-04

**Authors:** Hatice Beyazal Polat, Mehmet Beyazal, Fatma Beyazal Çeliker

**Affiliations:** ^1^Clinic of Internal Medicine, Medisina Van Hospital, Van, Turkey; ^2^Department of Radiology, School of Medicine, Yüzüncü Yıl University, Van, Turkey; ^3^Clinic of Radiology, Medisina Van Hospital, Van, Turkey

## Abstract

Acute acalculous cholecystitis and acute hemorrhagic cystitis due to Salmonella Typhi are a rare condition. A 24-year-old female patient was admitted to our clinic with abdominal pain, nausea, fever, headache, urinary burning, and bloody urine. Based on clinical, laboratory, and radiological evaluations, the patient was diagnosed with acute acalculous cholecystitis and acute hemorrhagic cystitis due to Salmonella Typhi. The patient was treated with intravenous ceftriaxone for two weeks. After the treatment, the patient's clinical and laboratory findings improved. Acute acalculous cholecystitis due to Salmonella Typhi concomitant with acute hemorrhagic cystitis is very rare and might be difficult to diagnose. Infectious agents such as Salmonella Typhi should be considered when acute acalculous cholecystitis and acute hemorrhagic cystitis are detected in adult patients with no underlying diseases.

## 1. Introduction

Salmonella Typhi are human-specific bacteria that are transmitted by the fecal-oral route. Acute Salmonella infections mainly present with gastrointestinal symptoms. Symptoms such as fever, abdominal pain, diarrhea, headache, cough, sore throat, and occasional constipation can be observed. Ten to fifteen percent of patients may develop complications. Acute acalculous cholecystitis and acute hemorrhagic cystitis due to Salmonella Typhi are a very rare clinical condition [1–4].

Here, we report a case of acute acalculous cholecystitis and acute hemorrhagic cystitis due to Salmonella Typhi.

## 2. Case Report

**Figure 1:**
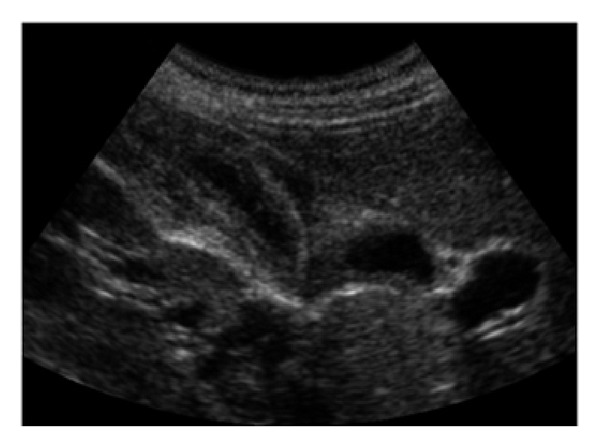
Ultrasound image. Thickening of the gallbladder wall. Minimal pericholecystic liquid is observed.

**Figure 2:**
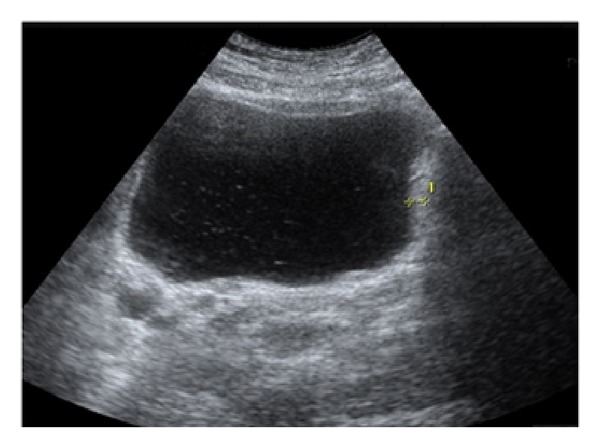
Thickening of the bladder wall and echogenicity in the bladder are present.

A twenty-four-year-old female patient was admitted to our clinic with the complaints of abdominal pain, nausea, fever, headache, urinary burning, and bloody urine. The patient had these symptoms for about one week with worsening of her symptoms over the previous few days. About two weeks prior she had diarrhea that lasted for about two days. The patient had no history of any illness or drug use. There were no confounding factors that could have contributed to the patient's urinary symptoms and hematuria (i.e., menses). The physical examination showed that the patient's general health was good. The patient's body temperature was 39°C, her pulse was 106/min, and her respiratory rate was 20/min. The abdominal examination showed tenderness in the right upper quadrant and suprapubic area. Laboratory examination disclosed white blood cell count of 21800/mm^3^ with 87% neutrophils, 8% monocytes, and 5% lymphocytes, platelet count of 199.000/mm^3^, and hemoglobin of 12.5 g/dL. The ratio of the alanine aminotransferase (ALT) to aspartate aminotransferase (AST) was 88/140 U/L (reference range: 7–5/19–48), the ratio of total to direct bilirubin was 5/3 mg/dL (reference range: 0.3–1.2 mg/dL), and the value for alkaline phosphatase was 300 U/L (reference range: 40–130 U/L) and for gamma-glutamyl transferase was 120 U/L (reference range: 7–60 U/L). The levels of serum amylase, lipase, creatinine, glucose, and electrolytes were within the normal range. Antibodies to hepatitis A, B, and C viruses and the human immunodeficiency virus were absent. C-reactive protein (CRP) was 179 mg/L, while the erythrocyte sedimentation rate was 22 mm/h. Urinalysis showed a specific gravity of 1.015, 75 erythrocytes per high-power field (HPF), and 12–15 leukocytes per HPF. Stool and blood cultures were negative. The urine cultures were positive for Salmonella Typhi. The patient had quantitative urine counts of 10^3^-10^4^ colony forming units/mL. There were no other culture results besides the positive urine for Salmonella. Salmonella O was found to be 1/200 in Gruber-Widal agglutination test. The chest X-ray did not show any abnormalities. Abdominal ultrasound examination showed diffuse thickening of the gallbladder wall and minimal pericholecystic fluid in the gallbladder. There were no calculi observed in the gallbladder lumen (Figure 1). There was an increase in the bladder wall thickness, and the bladder lumen had intense internal echoes which could be interpreted in favor of cystitis (Figure 2). The patient did not have any factors (i.e., diabetes mellitus, urolithiasis, urinary tract anomalia, etc.) predisposing to urinary tract infection. Based on these findings, the patient was diagnosed with acute cholecystitis and acute hemorrhagic cystitis due to Salmonella Typhi infection. The patient was started with 2 g of intravenous ceftriaxone and intravenous fluid per day. After 48 hours from the beginning of the treatment, the patient's temperature dropped to normal. On the sixth day of the treatment, the patient's complete blood count, serum biochemistry, CRP levels, and erythrocyte sedimentation ratio became normal. The patient was discharged on the 14th day upon completion of treatment.

## 3. Discussion

Typhoid fever is caused by the intracellular pathogen Salmonella Typhi, which continues to be a problem in many developing countries. Salmonella infections can cause acute gastroenteritis, enteric fever, and bacteremia. Moreover, the host can become a chronic carrier. Rarely, Salmonella infections may lead to nonintestinal infections such as cholecystitis, cystitis, pneumonia, appendicitis, hepatitis, osteomyelitis, myocarditis, spondylodiscitis, and meningitis. Extraintestinal salmonellosis generates approximately 8% of Salmonella episodes. Salmonella infections are more commonly seen in patients with chronic diseases, AIDS, and other immunocompromised patients and usually result in more severe disease in these patients [5, 6].

Acute acalculous cholecystitis accounts for approximately 5–10% of acute cholecystitis cases and is usually seen after burns, sepsis, multiple system failure, cardiovascular disease, diabetes, major operations, or prolonged parenteral hyperalimentation [7]. Typhoid fever, actinomycosis, parasitic infestations, and childhood diseases such as scarlet fever are among the rare causes of acalculous cholecystitis [8]. Acalculous cholecystitis due to Typhi is frequently reported in children in the endemic areas; however, it is rare in adults [9]. Abdominal pain, fever, vomiting, jaundice, and tenderness in the right upper quadrant are important symptoms of acute cholecystitis [1].

Acute hemorrhagic cystitis is rarely seen in healthy adults [10]. The etiology might be infectious agents, drugs, toxins, and radiation.* E. coli*, adenoviruses, and* Salmonella* can be important factors in the infections [11, 12]. Chronic diseases, immunosuppressive therapy, and urological anomalies can play an important role in the pathogenesis of urinary tract infections of* Salmonella* species [10]. Acute hemorrhagic cystitis manifests itself with urgency, frequent urination, dysuria, gross hematuria, and suprapubic sensitivity [13]. Although typhoid cystitis is rare, Salmonella can often be isolated from the urine during the course of typhoid fever [14]. This may suggest fecal contamination. However, in our case, suprapubic sensitivity and negative stool culture along with Salmonella isolation from the urine culture and presence of leukocytes and erythrocytes in the urine samples cystitis are due to Salmonella Typhi [15].

Diagnosis of Salmonella infections is usually made following the isolation of Salmonella. Blood cultures are positive in the early period. However, Salmonella can be cultured in 40–60% of these patients. Antibiotic therapy reduces the rate of culture growth. The Gruber-Widal agglutination test also contributes to the diagnosis [16].

An ultrasound examination can provide valuable information for the radiological evaluation of acute cholecystitis and acute hemorrhagic cystitis. The sensitivity of ultrasonography in acute cholecystitis varies between 67 and 92%. It is a good method to detect stones in the gallbladder. Thickening of the gallbladder wall detected by the ultrasound, the presence of pericholecystic fluid, and the absence of gallstones are the symptoms of acalculous cholecystitis [1]. However, the increase in diffuse thickness of the bladder wall is one of the symptoms of cystitis [15].

The treatment of typhoidal acute acalculous cholecystitis is controversial. Generally, if no complications such as gangrene or perforation of the gallbladder are present, conservative treatment is sufficient. Conservative treatment is followed by intravenous fluid and antibiotic therapy [1]. The patients with typhoidal acute hemorrhagic cystitis can be treated with cotrimoxazole, chloramphenicol, ceftriaxone, quinolones, and other antibiotics to which the microorganisms are sensitive [14, 17]. In our case, the cure was achieved by treatment with ceftriaxone along with intravenous fluids.

We presented a case of a combination of acalculous acute cholecystitis and acute hemorrhagic cystitis due to Salmonella in a patient with no known previous diseases. To the best of our knowledge, the combination of these two clinical cases has not been previously presented.

Extraintestinal manifestations due to Typhi are rarely seen and can be difficult to diagnose. Acalculous cholecystitis and/or hemorrhagic cystitis are rare complications caused by Salmonella Typhi. In the absence of predisposing factors in patients with acute acalculous cholecystitis and/or hemorrhagic cystitis, Salmonella Typhi should be considered as a cause, and diagnostic tests for Salmonella should be performed.

## References

[1] khan FY, Elouzi EB, Asif M (2009). Acute acalculous cholecystitis complicating typhoid fever in an adult patient: a case report and review of the literature. *Travel Medicine and Infectious Disease*.

[2] Wuthe HH, Aleksić S, Podschun R, Scheer-Sievers A (1992). Urinary tract infection due to a mucoid (M) form of Salmonella: a “new” transformation from M form into T1 form.. *Zentralblatt fur Bakteriologie*.

[3] Wilkins EG, Roberts C (1988). Extraintestinal salmonellosis. *Epidemiology and Infection*.

[4] Klotz SA, Jorgensen JH, Buckwold FJ, Craven PC (1984). Typhoid fever. An epidemic with remarkably few clinical signs and symptoms. *Archives of Internal Medicine*.

[5] Malik AS (2002). Complications of bacteriologically confirmed typhoid fever in children. *Journal of Tropical Pediatrics*.

[6] Rodríguez M, de Diego I, Mendoza MC (1998). Extraintestinal salmonellosis in a general hospital (1991 to 1996): relationships between Salmonella genomic groups and clinical presentations. *Journal of Clinical Microbiology*.

[7] Barie PS, Eachempati SR (2003). Acute acalculous cholecystitis. *Current Gastroenterology Reports*.

[8] Ruiz-Rebollo ML, Sánchez-Antolín G, García-Pajares F (2008). Acalculous cholecystitis due to Salmonella enteritidis. *World Journal of Gastroenterology*.

[9] Lai CH, Huang CK, Chin C, Lin HH, Chi CY, Chen HP (2006). Acute acalculous cholecystitis: a rare presentation of typhoid fever in adults. *Scandinavian Journal of Infectious Diseases*.

[10] Tena D, González-Praetorius A, Bisquert J (2007). Urinary tract infection due to non-typhoidal Salmonella: report of 19 cases. *Journal of Infection*.

[11] Loghman-Adham M, Tejero HT, London R (1988). Acute hemorrhagic cystitis due to *Escherichia coli*. *Child Nephrology and Urology*.

[12] Umekawa T, Kurita T (1996). Acute hemorrhagic cystitis by adenovirus type 11 with and without type 37 after kidney transplantation. *Urologia Internationalis*.

[13] Ramos JM, Aguado JM, García-Corbeira P, Alés JM, Soriano F (1996). Clinical spectrum of urinary tract infections due to nontyphoidal Salmonella species. *Clinical Infectious Diseases*.

[14] Arad E, Naschitz J, Yeshurun D (1996). Hemorrhagic cystitis as a presenting symptom of acute infection with Salmonella typhi. *Harefuah*.

[15] Lee HJ, Pyo JW, Choi EH (1996). Isolation of adenovirus type 7 from the urine of children with acute hemorrhagic cystitis. *Pediatric Infectious Disease Journal*.

[16] Kayserılı E, Hizarcioğlu M, Gülez P, Apa H, Keskın S (2005). [Co-existence of Kala-azar and salmonellosis in a 16 month-old baby? Or a false positive widal reaction in Kala-azar?]. *Turkiye Parazitoloji Dernegi*.

[17] McCarron B, Love WC (1997). Acalculous nontyphoidal salmonellal cholecystitis requiring surgical intervention despite ciprofloxacin therapy: report of three cases. *Clinical Infectious Diseases*.

